# Adsorption Characteristics of Modified Bamboo Charcoal on Cu(II) and Cd(II) in Water

**DOI:** 10.3390/toxics10120787

**Published:** 2022-12-14

**Authors:** Yizhuo Wang, He Li, Shaohua Lin

**Affiliations:** 1 School of Civil Engineering, Southeast University, Nanjing 210096, China; 2 School of Civil Engineering, Nanjing Forestry University, Nanjing 210037, China

**Keywords:** modified bamboo charcoal, adsorption, heavy metal fixation

## Abstract

With the development of industry in recent years, heavy metal contamination in water and substrate, which may pose a serious threat to human health if left untreated, has attracted increasing attention. Biochar is commonly used as an adsorbent/immobilizer for heavy metals in water and substrates because of its wide range of raw materials, low production cost, and good adsorption performance. In this paper, we selected abundant Moso bamboo as the raw material to make biochar (bamboo charcoal), modified bamboo charcoal using different methods to find the modified product with the best adsorption effect, assessed the adsorption performance of modified bamboo charcoal on Cu(II) and Cd(II) in solution, and investigated the effects of the solution concentration, adsorption time, pH, and temperature on the adsorption effect of KAM500-400-3 on Cu(II) and Cd(II). The effect of the solution concentration, adsorption time, pH, and temperature on the adsorption effect of KAM500-400-3 on Cu(II) and Cd(II) was investigated, and the adsorption mechanism of KAM500-400-3 on heavy metals Cu(II) and Cd(II) was analyzed by fitting the adsorption kinetics, adsorption isotherms, and adsorption thermodynamics. The adsorption/fixation characteristics of modified bamboo charcoal on heavy metals Cu(II) and Cd(II) in water and substrate were investigated. This study aimed to identify an effective material for the treatment of heavy metals in water and substrates and provide a reference for their application in practical engineering.

## 1. Introduction

With the continuous improvement in people’s day-to-day lives and industry production levels, the pollution of water and sediment by heavy metals is also increasing. At the same time, various sources of heavy metals are also increasing, mainly from industrial production such as the machinery processing industry, steelmaking, the nonferrous metal smelting industry, etc. In addition, automobile emissions, the disposal of waste batteries, and the abuse of agricultural fertilizers in daily life can cause serious heavy metal pollution in river bottom mud [1,2].

The composition of surface river and lake sediments is complex, mainly including clay, sediment, organic matter, and various mineral bodies [3,4]. Heavy metals in sediment mainly originate from polluted water bodies, and a series of transformation and migration processes such as adsorption–desorption, precipitation–dissolution, complexation–decomplexation, ion exchange, and redox occur between the sediment and the water body [5].

Because of their slow mobility and long residence time, heavy metals in the sediment are not degraded easily by microorganisms [6], and if they are not treated in time, they will become a “new source of pollution”, polluting the water body twice, poisoning the plants and animals in the water, and then further endangering human life and health through transmission via the food chain [7].

Prolonged exposure to heavy metal ions can cause serious harm to the human body, such as affecting the activity of enzymes, the nervous system, and some organs with detoxification functions. For example, in the 1950s and 1960s, when Japanese production mines exceeded the Cd(II) standard in nearby waters, some residents reported suffering from “bone pain”, a painful dent in the body caused by osteochondrosis [8]. The problem of how to treat heavy metals in river sediments and water bodies has become a pressing one [9].

Adsorption is the most commonly used method for treating heavy metals in water bodies and sediments, with the main benefits of being renewable, low cost, causing no "secondary pollution," and suitable for treating a wide range of wastewater concentrations. The specific adsorption mechanisms of biochar and modified biochar for heavy metals are shown in Table 1.

Biochar is a carbon-rich product obtained by charring biomass, and it has been widely studied for its advantages of low price and a wide range of sources. With its large porosity as well as specific surface area and the presence of many functional groups on its surface, it is an efficient and low-cost adsorbent and is often widely used to remove heavy metal pollutants from water [27]. Singh et al. conducted batch experiments on Cu(II), Cr(VI), Cd(II), and Pb(II) to investigate the effects of contact time, adsorbent dose, pH, and charring temperature on the adsorption of heavy metals. The experimental results showed that the adsorption capacity was positively correlated with the adsorption time in the first 20 min but gradually tended to reach equilibrium after 20 min. The adsorbent dose and pyrolysis temperature were positively correlated with the removal effect of biochar for heavy metals, and the maximum removal efficiency of biochar could reach 99.86% at pH = 4 [28]. Park et al. investigated the adsorption of heavy metals on sesame straw charcoal (SSB) and compared its adsorption of single and multiple metals. They found that SSB had superior adsorption effects on heavy metals, and the adsorption capacity of SSB for single heavy metals were, in descending order, Pb(II) > Cu(II) > Cr(VI) > Zn(II) > Cd(II), and for polymetals were, in descending order, Pb(II) > Cd(II) > Cr(VI) > Cu(II). The adsorption capacity for polymetallics was much greater than for monometallics [29].

Although biochar has a certain adsorption effect on heavy metals, the adsorption capacity is relatively limited, and, in order to improve the utilization of biochar, biochar should be further modified to effectively enhance its remediation function. Modified biochar can compensate for some of the shortcomings of single biochar and improve the adsorption effect, so modified biochar materials have received much attention in recent years [30].

There are usually two types of modifying biochar: one is to modify the prepared biochar by impregnation with chemicals or co-precipitation with metals; the other is to prepare biomass raw materials by mixing them with modification reagents and preparing them by high-temperature pyrolysis. Biochar modification includes surface structure modification and surface chemical modification. Surface structure modification is mainly to change the pore structure of biochar to increase the specific surface area in order to increase the adsorption capacity. Surface chemical modification includes redox modification, acid-base surface modification, adsorbent compound modification, and activation modification. These methods can improve the adsorption capacity of biochar for heavy metals by increasing the number of functional groups on the surface of the biochar. The general preparation process of mixing biomass raw materials with other reagents for pyrolysis modification includes mixing, filtration, drying, pyrolysis, and debinding [31].

In this study, the adsorption characteristics of optimally modified bamboo char KAM500-400-3 for heavy metals Cu(II) and Cd(II) were investigated by static adsorption experiments. The effects of solution concentration, adsorption time, pH, and temperature on the adsorption effect of KAM500-400-3 on Cu(II) and Cd(II) were investigated in the static adsorption experiments, and the adsorption mechanisms of KAM500-400-3 on Cu(II) and Cd(II) were analyzed by means of adsorption kinetics, adsorption isotherms, and adsorption thermodynamics. The adsorption mechanism of KAM500-400-3 for Cu(II) and Cd(II) was analyzed by fitting the adsorption kinetics, adsorption isotherms, and adsorption thermodynamics.

## 2. Materials and Methods

### 2.1. Main Experimental Apparatus and Materials

The selected biomass material in this experiment is *Phyllostachys pubescens*, which is from a bamboo forest in Jurong, Jiangsu Province. Table 2 lists the experimental instruments, instrument specifications, and applications required for this experiment. Table 3 lists the reagents, specifications, and purity required for this experiment. The water used in this experiment is ultrapure water (the resistivity is 18.25 MQ·cm) [32].

### 2.2. Preparation Method of Bamboo Charcoal

Moso bamboo, retrieved from a bamboo forest in Jurong, was washed with tap water and deionized water and dried naturally. The Moso bamboo was dried in a constant-temperature drying oven at 80 °C after being cut into small pieces with a chainsaw [33]. The dried bamboo pieces were cut into strips with branch shears and ground to a powder with a 100 mesh sieve to obtain 100 mesh bamboo powder in a self-sealing bag. A quartz tube was filled with 100 g of 100 mesh bamboo powder and placed in a tube furnace. We set the charring temperature of the tube furnace to 400 °C, 500 °C, 600 °C, or 700 °C. The heating procedure was 10 °C per minute, and the temperature was kept at the set temperature for an hour and then gradually cooled down [34]. After the temperature was lowered to room temperature, the Moso bamboo biochar was taken out and stored in sealed bags, which were labeled BC400, BC500, BC600, and BC700 according to the charring temperature.

Please see the Preparation methods of modified bamboo charcoal, Analysis of surface morphology and element distribution of bamboo charcoal, Specific surface area determination, FT-IR analysis, Adsorption experiments of bamboo charcoal and modified bamboo charcoal on heavy metals Cu(II) and Cd(II) in the Supplementary Material.

## 3. Results

### 3.1. Effect of Charring Temperature

**Figure 1:**
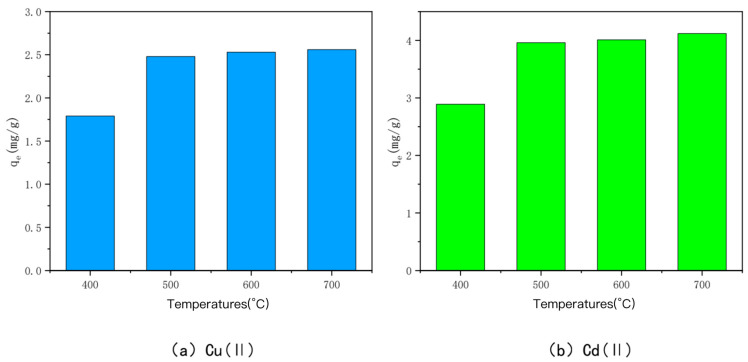
The adsorption capacity of Cu(II)/Cd(II) adsorbed by bamboo charcoal at different carbonization temperatures.

The effect of charring temperature on the ability of bamboo char to adsorb heavy metals Cu(II) and Cd(II) is shown in Figure 1. The bamboo charcoal fired at the charring temperatures of 400 °C, 500 °C, 600 °C, and 700 °C was recorded as BC400, BC500, BC600, and BC700, respectively. The initial concentrations of heavy metals Cu(II) and Cd(II) in the experiments were all 50 mg/L, the pH was 6, and the volumes added were all 120 mL at an ambient temperature of 25 °C. The adsorption amounts of BC400, BC500, BC600, and BC700 were 1.79 mg/g, 2.48 mg/g, 2.53 mg/g, and 2.56 mg/g for Cu(II) and 2.89 mg/g, 3.96 mg/g, and 4.01 mg/g for Cd(II), respectively. The adsorption amounts of BC400 were significantly smaller than those of BC500, BC600, and BC700, and the adsorption amounts of the latter three were not significantly different. The results showed that when the charring temperature was lower, the adsorption amounts of Cu(II) and Cd(II) were smaller, and when the charring temperature was higher than 500 °C, the adsorption amounts of bam-boo char increased, but the increment was smaller. The reason behind this phenomenon is that, with the increasing carbonization temperature, the functional groups on the surface of bamboo charcoal will be more evenly distributed. In addition, the increase in carbonization temperature will create a more abundant pore structure for activated carbon, and the increase in the specific surface area of carbon will help to increase the adsorption capacity. The adsorption capacities of BC600 and BC700 were only 2.02% and 3.22% higher than those of BC500 for heavy metal Cu, and 1.26% and 4.04% higher than those of BC500 for heavy metal Cd. Considering the time cost and energy consumption for the preparation of the three in the tube furnace, BC500 was selected for the following modification experiments.

### 3.2. Effects of Different Modification Methods

(1) KOH-modified bamboo charcoal

**Figure 2:**
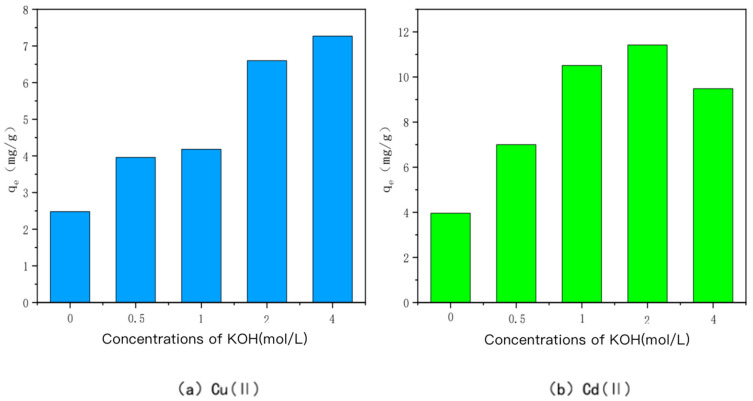
The adsorption capacity of Cu(II)/Cd(II) adsorbed by KOH-modified carbon at different concentrations of KOH.

The ability of KOH-modified bamboo charcoal to adsorb Cu(II) and Cd(II) at different concentrations is shown in Figure 2. KOH-modified bamboo charcoal was labeled as KBC500-0.5, KBC500-1, KBC500-2, and KBC500-4 in descending order according to the concentration of KOH solution mixed with it: 0.5 mol/L, 1 mol/L, 2 mol/L, or 4 mol/L. Heavy metal Cu(I) adsorption amounts of KBC500-0.5, KBC500-1, KBC500-2, and KBC500-4 were 3.96 mg/g, 4.18 mg/g, 6.60 mg/g, and 7.27 mg/g, respectively, whereas heavy metal Cd(II) adsorption amounts were 7.00 mg/g, 10.51 mg/g, and 10.51 mg/g, respectively. The adsorption amounts of unmodified BC-500 for Cu(II) and Cd(II) were 2.48 mg/g and 3.96 mg/g, respectively, which could be attributed to the stronger adsorption capacity of KOH-modified carbon for heavy metals Cu(II) and Cd(II). The specific surface area of bamboo char increased after alkali treatment, as did the type and number of oxygen-containing functional groups on the surface, enhancing its effect on the adsorption of heavy metals Cu(II) and Cd(II) [35]. The growth rate of the adsorption capacity of charcoal reached its maximum when the concentration of KOH was 2 mol/L. The growth efficiency of the adsorption capacity of charcoal for Cu(II) decreased when the concentration was greater than 2 mol/L, and the adsorption amount of Cd(II) decreased as well, probably because the KOH solution neutralized the acidic oxygen-containing functional groups on the surface of BC500, resulting in a decrease in acidic oxygen-containing functional groups and an increase in basic oxygen-containing functional groups [36,37,38,39]. The KOH solution will cause the etching effect of BC500: as its concentration increases, new pores will form on the surface, the original pores on the surface will gradually increase, and the specific surface area will also increase [40]. However, when the alkali concentration is too high, the acidic oxygen-containing functional groups on the surface of BC500 are consumed, resulting in a slow increase or even a decrease in the specific surface area of BC500 [41,42,43]. In summary, KBC500-2 has the best adsorption capacity for heavy metals Cu(II) and Cd(II) when the KOH concentration is 2 mol/L.

(2) KOH-activated bamboo charcoal
  ① Charcoal-to-alkali ratio

**Figure 3:**
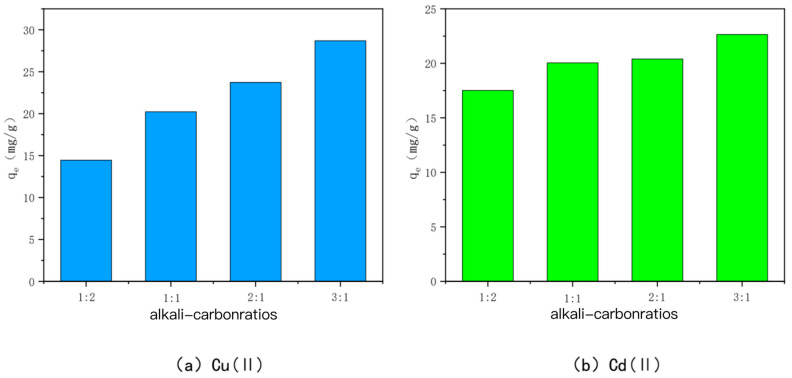
The adsorption capacity of Cu(II)/Cd(II) by KOH-activated modified carbon at various alkali-to-carbon ratios.

The adsorption amounts of Cu(II) and Cd(II) adsorbed by KOH-modified carbon with different alkali–carbon ratios are shown in Figure 3. For 700-3, the adsorption amounts were 14.45 mg/g, 20.23 mg/g, 23.73 mg/g, and 28.70 mg/g for Cu(II), and 17.51 mg/g, 20.05 mg/g, 20.39 mg/g, and 22.65 mg/g for Cd(II), respectively, which shows that when the ratio of KOH to BC500 is larger, the effect of KOH activation modification is good. This may be because, when the alkali-to-carbon ratio is small, KOH only reacts with the surface phase of BC500 and cannot form a large number of micropores and mesopores [44]. When the alkali-to-carbon ratio is further expanded, KOH can enter the graphite layer of the carbon and react with the inner carbon, consuming a large amount of carbon to produce carbon monoxide and hydrogen, which makes the pore volume of the carbon and the number of mesopores and micropores increase. In summary, when the alkali carbon ratio is 3:1, the best adsorption effects for Cu(II) and Cd(II) are obtained.

② Temperature

**Figure 4:**
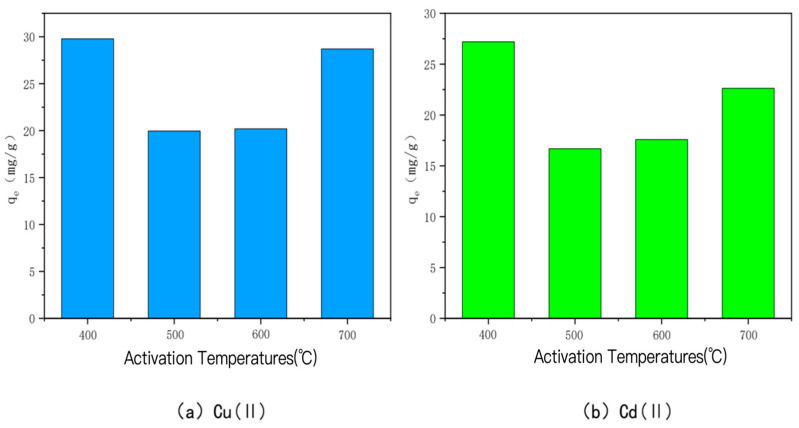
The adsorption capacity of Cu(II)/Cd(II) adsorbed by KOH-activated modified carbon at different activation temperatures.

The amounts of Cu(II) and Cd(II) adsorbed by KOH-activated modified bamboo charcoal at different activation temperatures are shown in Figure 4. The adsorption amounts of Cu(II) and Cd(II) were 29.79 mg/g, 19.97 mg/g, 20.20 mg/g, and 20.20 mg/g, respectively, when the activation temperature was 400 °C, 500 °C, 600 °C, and 700 °C. The adsorption amounts for Cu(II) were 29.79 mg/g, 19.97 mg/g, 20.20 mg/g, and 28.70 mg/g, and for Cd(II), they were 27.20 mg/g, 16.69 mg/g, 17.59 mg/g, and 22.65 mg/g, respectively, when the activation temperature was 400 °C. The adsorption of Cd(II) and Cu(II) was the highest. This may be due to the fact that increasing the activation temperature at a high alkali-to-carbon ratio causes significant ablation of the carbon, which reduces the active carbon spots on the surface and consequently decreases the adsorption performance of the carbon [45,46]. A similar phenomenon has been observed, i.e., the saturation adsorption capacity of the char is roughly negatively correlated with the activation temperature when the alkali-to-carbon ratio is too high, and the larger the alkali-to-carbon ratio, the more obvious the phenomenon is.

(3) HNO_3_-modified bamboo charcoal

**Figure 5:**
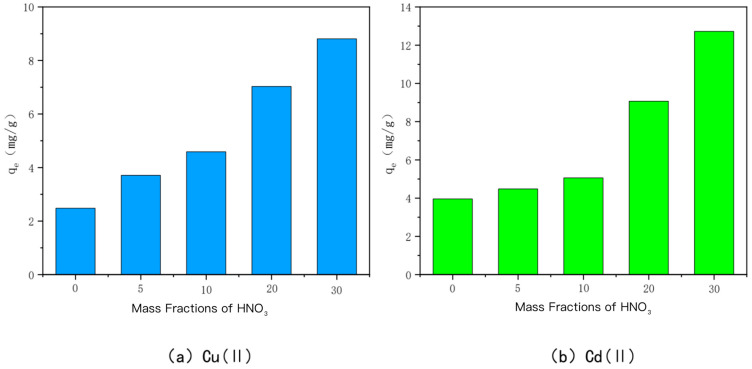
The adsorption capacity of Cu(II)/Cd(II) adsorbed by HNO_3_-modified carbon at different mass fractions of HNO_3_.

The ability of HNO_3_-modified charcoal to adsorb Cu(II) and Cd(II) at different concentrations is shown in Figure 5. HNO_3_-modified bamboo charcoal is labeled as HBC500-5, HBC500-10, HBC00-20, and HBC500-30, in descending order of the mass fraction of HNO_3_ solution mixed with it at 5%, 10%, 20%, and 30%, respectively. The adsorption amounts of HBC500-5, HBC500-10, HBC00-20, and HBC500-30 for heavy metal Cu(II) were 3.71 mg/g, 4.59 mg/g, 7.03 mg/g, and 8.81 mg/g, respectively, and the adsorption amounts of heavy metal Cd(II) were 4.48 mg/g, 5.06 mg/g, 9.07 mg/g, and 12.07 mg/g, respectively. The adsorption capacities of unmodified BC500 for Cu(II) and Cd(II) were 2.48 mg/g and 3.96 mg/g, respectively, and the adsorption capacities of HNO3-modified bamboo charcoal increased with increasing HNO3 mass fraction. The adsorption capacity of bamboo charcoal for Cu(II) and Cd(II) gradually increased, and the strongest adsorption capacity was achieved when the mass fraction of HNO_3_ was 30%, i.e., HBC500-30. This may be due to the fact that the number of oxygenated acidic functional groups, such as carboxyl groups, on the surface of BC500 gradually increased with the increase in the mass fraction of HNO_3_ and it was also experimentally confirmed that the porosity and specific surface area of the carbon increased significantly when the mass fraction of HNO_3_ was 30%.

### 3.3. Characterization of Modified Bamboo Charcoal

#### 3.3.1. Morphological Analysis

**Figure 6:**
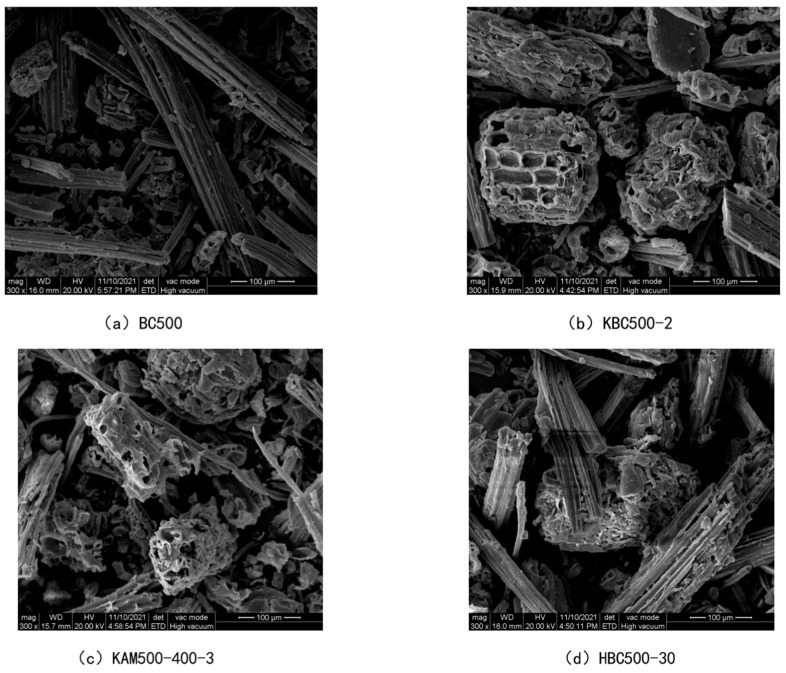
SEM images of bamboo charcoal and modified bamboo charcoal.

The surface morphologies of bamboo charcoal, as well as modified bamboo charcoal, are shown in Figure 6. Figure 6a–d shows the surface morphologies of unmodified BC500, KBC500-2-, HBC500-30-, and KAM500-400-3-modified charcoal, respectively. As can be seen from Figure 6, the surface of unmodified BC500 is smoother, and the char is mainly tubular in shape with a relatively intact structure. The surface of KBC-500-2 modified by KOH alkali obviously became very rough; a large number of round and oval collapses appeared, and the distribution was more uniform. The morphology was similar to that of honeycomb, which indicated that KOH would cause the char to generate a pore structure or even break the char structure [47,48,49]. The shape of HBC500-30 modified by HNO_3_ is similar to that of unmodified BC500—parallel tubular bundles—but the surface appears scaly and relatively rough. The surface of KAM500-400-3 after KOH activation showed a lot of collapse and unevenness; its structure was far from that of BC500, and large pores were clearly visible on the cross section.

#### 3.3.2. Measurement of Specific Surface Area

The porous structure parameters of bamboo charcoal and modified bamboo charcoal are shown in Table 4. Generally speaking, the specific surface area, total pore capacity, and average pore diameter of carbon will increase after acid–base modification, but the detected results are inconsistent with the expected situation. These parameters, KBC500-2 and HBC500-30, are lower than the unmodified BC500 in destroying the structure of the carbon surface, which is the same as the SEM. The specific surface area of KAM500-400-3 is 1.45 times that of BC500, the total pore capacity is 4.57 times that of BC500, and the average aperture is 5.16 times that of BC500. These values are also much higher than the porous structural parameters of bamboo charcoal and modified bamboo charcoal. The specific surface area, pore size, and pore capacity of the carbon surface can effectively increase the adsorption capacity of heavy metals Cu(II) and Cd(II) [50].

#### 3.3.3. Surface Functional Group Determination

**Figure 7:**
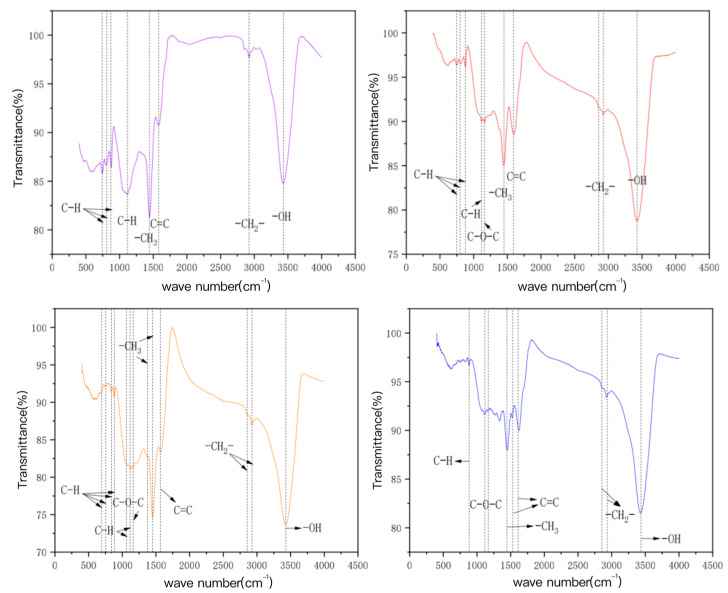
The FT—IR map of the bamboo charcoal and the modified bamboo charcoal.

The characteristic FT-IR spectra peaks of bamboo charcoal and bamboo charcoal-modified charcoal, as shown in Figure 7 for BC500, are mainly aromatic hydrocarbons (C–H and C = C), fatty hydrocarbons (–CH_x_), hydroxyl groups (–OH), etc. The characteristic spectrum peak of KBC500-2 increases the oxygen-containing functional group (C–O–C) compared with BC500, and its vibration mode is aromatic ether expansion vibration. HBC500-30 contains fewer aromatic hydrocarbons (C-H) than BC500, probably because HNO3 is strongly oxidized as a strong acid, oxidizing some unstable functional groups and neutralizing them with the alkaline functional groups on the carbon surface [51,52,53,54,55]. In addition, HBC500-30 forms an aromatic hydrocarbon (C = C), an oxygen-containing functional group (C–O–C), and a fatty hydrocarbon (–CH_2_−), whose vibration modes are aromatic ring skeleton vibration, aromatic ether expansion vibration, and symmetry extension vibration of the long-chain methylene group, respectively. Compared with BC500, KAM500-400-3 has significantly more absorption peaks, including aromatic hydrocarbon (C–H), oxygen-containing functional group (C–O–C), adipose C hydrocarbon (–CH_x_), etc. The vibration modes are respectively the out of plane bending vibration of aromatic ring C-H, the stretching vibration of aromatic ether, the in-plane bending vibration of aromatic ring C-H, and the antisymmetric stretching vibration of long chain methylene, which further explains the reason why the KOH activated carbon has strong adsorption capacity for heavy metals Cu (II) and Cd (II). [56,57].

#### 3.3.4. Element Distribution

The distribution of the elements before and after the adsorption of heavy metals Cu(II) and Cd(II) is shown in Table 5. BC500, KAM500-400-3, KAM500-400-3, and HBC500-30, after the adsorption of Cu(II), had a percentage content of 0.27%, 1.62%, 7.77%, and 0.41%, respectively. The percentage content of the adsorbed Cd(II) was 1.36%, 1.61%, 8.42%, and 1.70%, respectively. This further proves that the adsorption capacity of the modified carbon for the heavy metals Cu(II) and Cd(II) will be improved. The three modification methods had the following effects: bamboo charcoal modified by HNO_3_ and KOH had a better adsorption effect than unmodified bamboo charcoal, but the improvement in adsorption performance was not significant; and the heavy metal Cu(II) was adsorbed after modification by KOH activation. The capacity of Cd(II) was significantly enhanced, as shown by the results of the above adsorption experiments.

In this section, the effect of charring temperature on the adsorption capacity of bamboo char for heavy metals Cu(II) and Cd(II) is investigated, and the char with optimal charring temperature is modified with KOH, activated with KOH, and modified with HNO_3_, respectively. The results were as follows.

The adsorption capacity of bamboo char for the heavy metals Cu(II) and Cd(II) was proportional to the charring temperature, i.e., the adsorption capacity of bamboo char increased as the charring temperature increased gradually. The adsorption capacity of BC400 was significantly smaller than that of BC500, BC600, and BC700, and the difference between the adsorption capacities of the latter three was not significant [58,59,60,61]. The adsorption capacities of BC600 and BC700 for heavy metals Cu(II) were only 2.02% and 3.22% higher than those of BC500, respectively, and 1.26% and 4.04% higher than those of BC500 for heavy metal Cd(II); considering the time cost and energy consumption of the three tube furnace preparations, BC500 was selected for the subsequent modification experiments [62].

The KOH-modified, KOH-activated modified, and HNO_3_-modified bamboo charcoals were better than the unmodified ones in terms of the adsorption of heavy metals Cu(II) and Cd(II) [63]. Among them, the optimal conditions for KOH modification were a KOH concentration of 2 mol/L, KOH activation modification at an alkali-to-carbon ratio of 3:1, and an activation temperature of 400 °C. The best adsorption capacity of HNO_3_ modification, with a mass fraction of HNO_3_ of 30% for heavy metals Cu(II) and Cd(II), i.e., KBC500-2, KAM500-400-3, and HBC500-30, indicated the best modified bamboo charcoal for the three modification methods. Among the three, KAM500-400-3 had the highest adsorption capacity for heavy metals Cu(II) and Cd(II) [64,65,66].

The morphology of the modified char surface became relatively rough compared with that before the modification, and a large number of pores appeared. The specific surface area, total pore volume, and average pore size of the acid–base-modified bamboo char were smaller than those of the unmodified bamboo char, while the specific surface area of KAM500-400-3 was 1.45 times that of BC500, the total pore volume was 4.57 times that of BC500, and the average pore size was 5.16 times that of BC500. Meanwhile, the number and types of functional groups on the surface of the modified carbon were significantly increased, among which the number of absorption peaks of KAM500-400-3 increased the most. The results of an elemental analysis showed that the proportion of O elements in the bamboo char modified by HNO_3_ and KOH activation increased significantly, and the proportion of Cu and Cd elements in the modified bamboo char with adsorbed Cu(II) and Cd(II) was the largest in KAM500-400-3 [67,68].

In summary, when the conditions of KOH activation modification were a 3:1 alkali-to-carbon ratio and 400 °C activation temperature, KAM500-400-3 had the strongest adsorption capacity for heavy metals Cu(II) and Cd(II) among all modified bamboo charcoals, so it was selected for the adsorption characteristics study.

### 3.4. Effect of Solution Concentration 

**Figure 8:**
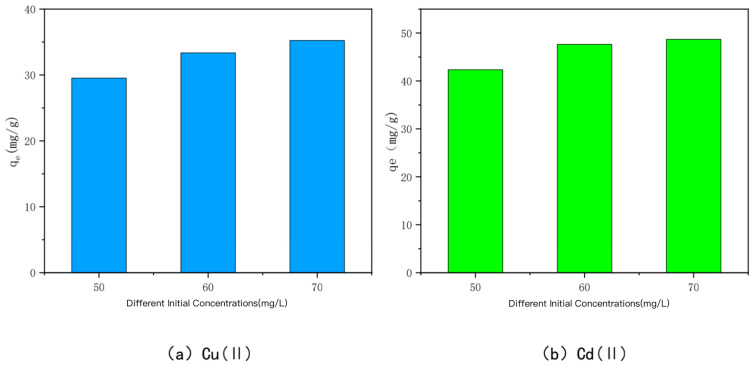
Effect of different initial concentrations on the adsorption of Cu(II /Cd(II) by KAM500-400-3.

The relationship between the adsorption amount of KAM500-400-3 on Cu(II) and Cd(II) and the initial concentrations of Cu(II) and Cd(II) is shown in Figure 8. When the initial concentrations of Cu(II) and Cd(II) were 50 mg/L, 60 mg/L, and 70 mg/L, the adsorption amounts of KAM500-400-3 were 29.53 mg/g, 33.36 mg/g, and 35.24 mg/g for Cu(II) and 42.35 mg/g, 47.65 mg/g, and 48.68 mg/g for Cd(II), respectively. From the experimental results, it can be seen that the adsorption of Cu(II) and Cd(II) by the optimal modified effect carbon increased with the increase in the initial concentration, but the adsorption amount leveled off after reaching a certain adsorption time because the driving force to overcome the mass transfer resistance improved with the increase in the initial concentration [69,70,71,72,73]. When the initial solution of Cu(II) and Cd(II) is at a low concentration, the number of heavy metal Cu(II) and Cd(II) ions is limited. KAM500-400-3 has sufficient surface adsorption sites and functional groups, and a large number of active sites compete for the limited Cu(II) and Cd(II) ions. Cu(II) and Cd(II) ions and carbon adsorption can be produced rapidly, but the adsorption amount of KAM500-400-3 for heavy metals Cu(II) and Cd(II) is not high because the adsorption sites are not fully utilized. As the initial concentration increases, the concentration gradient between KAM500-400-3 and the solution interface increases, causing Cu(II) and Cd(II) to migrate from the solution to the solid surface, and a large amount of free Cu(II) and Cd(II) compete for the limited adsorption sites and functional groups on the surface of KAM500-400-3, causing it to gradually reach the adsorption saturation state. The excess of the optimal modified carbon at the initial concentration is converted into an excess of heavy metals Cu(II) and Cd(II), the adsorption amount does not increase indefinitely once it reaches a certain value.

### 3.5. Effect of Solution pH

**Figure 9:**
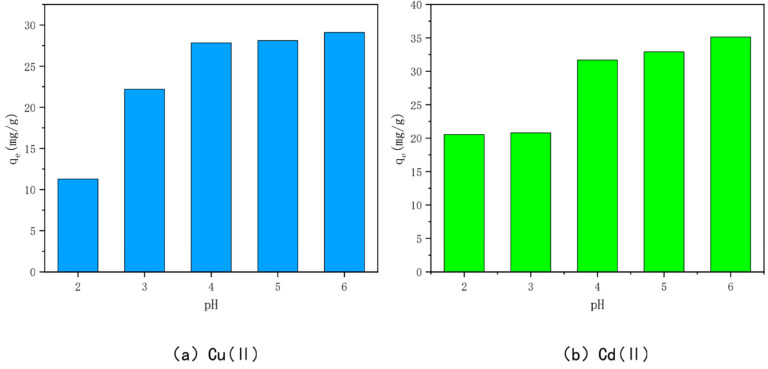
Effect of pH on the adsorption of Cu(II)/Cd(II) by KAM500-400-3.

The effects of different initial solution pH on the adsorption of Cu(II) and Cd(II) by the optimally modified bamboo charcoal are shown in Figure 9. When the pH is 2, the adsorption of Cu(II) and Cd(II) by the optimally modified bamboo charcoal is the smallest—11.28 mg/g and 20.54 mg/g, respectively. This indicates that a lower pH is not ideal for the adsorption of Cu(II) and Cd(II) by optimally modified bamboo charcoal, but the adsorption of Cu(II) and Cd(II) increased with an increase in solution pH (pH < 7). This is because the solution pH can change the morphology of heavy metals and affect the degree of protonation and deprotonation of functional groups on the surface of optimally modified bamboo charcoal [74,75]. When the pH of the solution is small and acidic, the ionic forms of Cu(II) and Cd(II) in the solution are primarily Cu(II), Cu(OH)^+^/Cd(II), and Cd(OH)^+^, and the H^+^ in the solution will electrostatically repel them and compete for the active sites on the char surface together, inhibiting the adsorption of Cu(II) and Cd(II) on the char. 

When the pH of the solution gradually increases, OH^−^ will be produced in the solution, and its amount will increase with the increase of pH.OH^−^ can increase the number of negative charges on the carbon surface so as to attract cations such as Cu (II) and Cd (II). At this time, Cu (II), Cu (OH)^+^/Cd (II), Cd (OH)^+^and Cu (OH)_2_/Cd (OH)_2_ precipitates will appear in the solution. However, when the pH value increases further, on the one hand, OH^−^ will compete with Cu(II) and Cd(II) for the oxygen-containing groups on the surface of the optimally modified bamboo carbon, which will reduce the adsorption capacity; on the other hand, some precipitation reactions will occur with Cu(II) and Cd(II) to form strong oxidizing precipitates, which will inhibit the adsorption of the optimally modified bamboo carbon, and thus, the adsorption capacity will be reduced accordingly [76,77,78].

### 3.6. Effect of Adsorption Time and Adsorption Kinetics

**Figure 10:**
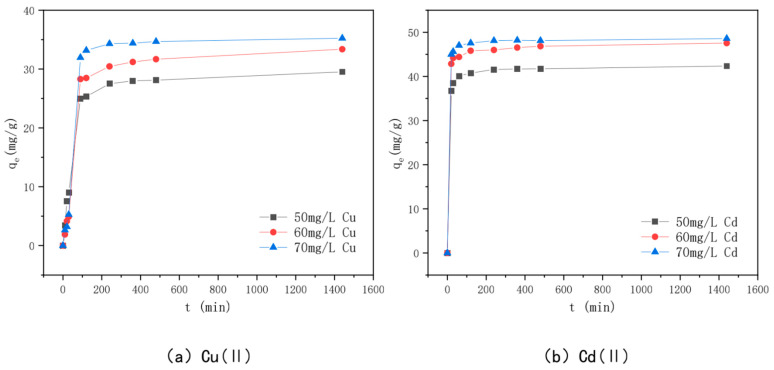
Effect of adsorption time on the adsorption of different concentrations of Cu(II)/Cd(II).

The relationship between adsorption time and adsorption amounts of Cu(II) and Cd(II) by KAM500-400-3 is shown in Figure 10. The figure shows that when the Cu(II) and Cd(II) solution concentrations are 50 mg/L, 60 mg/L, and 70 mg/L, respectively, the adsorption time follows approximately the same trend as the adsorption amounts. The adsorption of Cu(II) by KAM500-400-3 increased rapidly in the first two hours of adsorption, increased slowly towards the adsorption equilibrium, and finally reached 29.53 mg/g, 33.36 mg/g, and 35.34 mg/g for Cd(II), respectively. The adsorption amount increased significantly in the first hour of adsorption and then slowly tended to the adsorption equilibrium. Because the adsorption sites, pore structure, and functional groups on the surface of KAM500-400-3 are sufficient at the start of adsorption, the Cu(II) and Cd(II) adsorption amounts change rapidly [79]. However, after a period of adsorption, the adsorption sites on the surface of KAM500-400-3 are gradually occupied by heavy metal ions, the pore structure is further filled, the functional groups form complexes with heavy metal ions to stabilize the carbon surface, and the adsorption rate decreases because the concentration of heavy metal ions in the solution decreases at this time. Furthermore, because the surface of KAM500-400-3 is filled with Cu(II)/Cd(II) charges, and the Cu(II)/Cd(II) charges in the solution produce repulsion, making further adsorption difficult, with the increase in adsorption time, the adsorption of KAM500-400-3 for Cu(II) and Cd(II) tends to plateau after reaching a certain amount [80].

**Figure 11:**
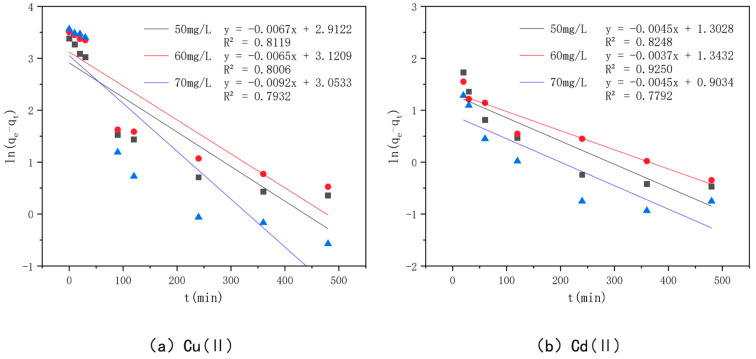
Pseudo-first-order kinetic models of different concentrations of Cu(II)/Cd(II).

**Figure 12:**
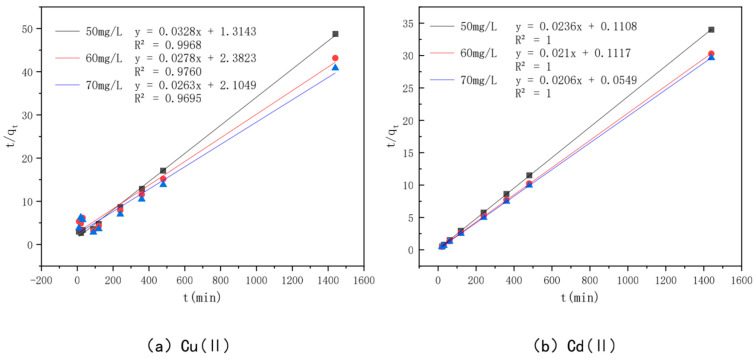
Pseudo-second-order kinetic models of different concentrations of Cu(II)/Cd(II).

In order to investigate the adsorption mechanism of Cu(II) and Cd(II) by the optimal effect modified bamboo charcoal, the proposed primary kinetic model shown in Figure 11 and the proposed secondary kinetic model shown in Figure 12 were introduced in this experiment to investigate the adsorption process, and the fitted data of the kinetic model are shown in Table 6. The *R^2^* values of modified bamboo charcoal with optimal effect for adsorption of Cu(II) at concentrations of 50 mg/L, 60 mg/L, and 70 mg/L were 0.8119, 0.8006, and 0.7932, respectively, and the *R*^2^ values for adsorption of Cd(II) at concentrations of 50 mg/L, 60 mg/L, and 70 mg/L were 0.8248, 0.9250, and 0.732, respectively. In the proposed secondary kinetic model, the *R*^2^ values of modified bamboo carbon were 0.9968, 0.9760, and 0.9695 for the adsorption of Cu(II) at 50 mg/L, 60 mg/L, and 70 mg/L, respectively, and the *R*^2^ values of Cd(II) at 50 mg/L, 60 mg/L, and 70 mg/L were 1, 2, and 3, respectively. When the proposed secondary kinetics were combined with the above experimental results, they fitted better than the proposed primary kinetics. This indicates that chemisorption and physical adsorption occur simultaneously in the adsorption of Cu(II) and Cd(II) by KAM500-400-3, with chemisorption being dominant. This chemisorption involves electron covalency or electron migration between the adsorbed material and the adsorbent, and physical adsorption such as precipitation and ion exchange may also exist. According to the kinetic time, equilibrium can be reached in about 6 h for Cu(II) adsorption and 4 h for Cd(II) adsorption by KAM500-400-3, which can be used to determine the adsorption time for subsequent experiments.

**Figure 13:**
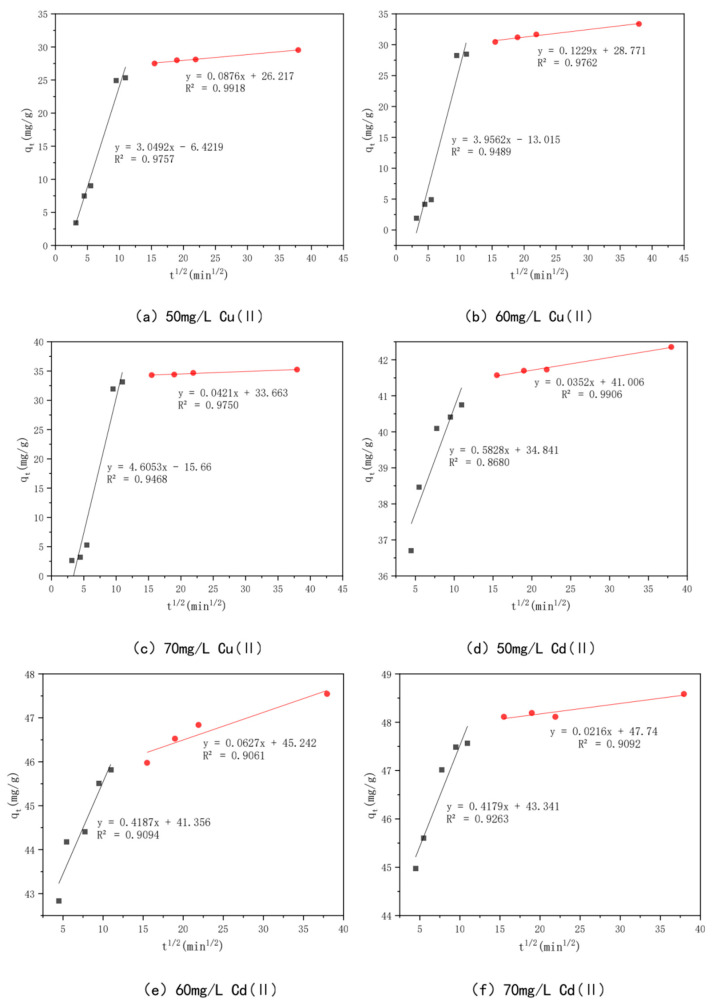
Intraparticle diffusion models of different concentrations of Cu(II)/Cd(II).

This experiment employs an intraparticle diffusion model to further analyze the data in order to better understand the diffusion mechanism of Cu(II) and Cd(II) on KAM500-400-3, as shown in Figure 13. The intraparticle diffusion process mainly consists of three steps: mass transfer, adsorption, and diffusion. The intraparticle diffusion process of Cu(II) and Cd(II) on KAM500-400-3 has similar fitting results. In this study, the adsorption process (first stage) and diffusion process (second stage) are the main steps of intraparticle diffusion because the mass transfer process is completed in a very short time [81]. The slopes (kp_1_ and kp_2_) of the first and second stages for Cu(II) were 3.0492, 0.0876, 3.9562, 0.1229, and 4.6053, 0.0421, respectively, with KAM500-400-3 adsorption concentrations of 50 mg/L, 60 mg/L, and 70 mg/L. The correlation coefficients *R*_1_^2^ were 0.9757, 0.9489, and 0.9468, and the *R^2^* values were 0.9918, 0.9762, and 0.9750, respectively. For Cd(II) with KAM500-400-3 adsorption concentrations of 50 mg/L, 60 mg/L, and 70 mg/L, the slopes of the first and second phases (kp_1_ and kp_2_) were 0.5828, 0.0352, 0.4187, 0.0627, and 0.4179, 0.0216, respectively. The correlation coefficients *R*_1_^2^ were 0.8680, 0.9094, and 0.9263, and the *R*^2^ values were 0.9906, 0.9061, and 0.9092, respectively, indicating that Cu(II) and Cd(II) on KAM500-400-3 were consistent with the intraparticle diffusion model. The adsorption rates of Cu(II) and Cd(II) in the pores of KAM500-400-3 are higher than their diffusion rates in the particles. At the same time, the fitted lines of both did not cross the origin, signifying the existence of other rate control steps besides intraparticle diffusion.

### 3.7. Adsorption Isotherms

**Figure 14:**
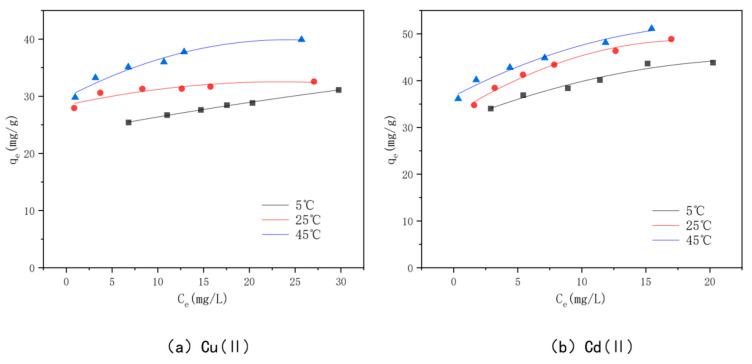
The KAM500-400-3 isothermal adsorption line adsorbing Cu(II)/Cd(II) at different temperatures.

The adsorption isotherms for Cu(II) and Cd(II) adsorption at different temperatures are shown in Figure 14. The adsorption amount of the modified bamboo charcoal increases continuously for these two heavy metals as the volume of Cu(II) solution increases from 60 to 160 mL and the volume of Cd(II) solution increases from 70 to 140 mL. The adsorption of Cu(II) and Cd(II) by modified bamboo charcoal increased with increasing equilibrium concentration, but the increase in adsorption decreased gradually [82]. At temperatures of 5 °C, 25 °C, and 45 °C, the adsorption amounts of optimally modified bamboo charcoal reached 31.12 mg/g, 32.57 mg/g, and 39.91 mg/g for Cu(II), and 43.83 mg/g, 48.00 mg/g, and 51.00 mg/g for Cd(II), when the volume of Cd(II) increased from 60 to 160 mL, respectively. According to the experimental results, the optimal modified bamboo charcoal is more effective at treating a large amount of Cu(II) and Cd(II) solution with a higher concentration and has a better adsorption effect on Cd(II) than Cu(II) [83].

**Figure 15:**
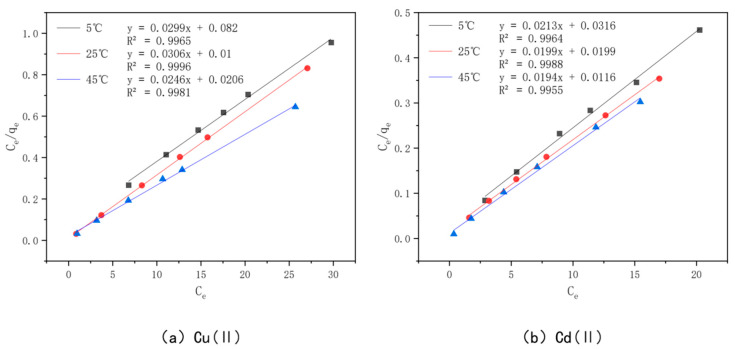
Langmuir models of KAM500-400-3 adsorbing Cu(II)/Cd(II) at different temperatures.

**Figure 16:**
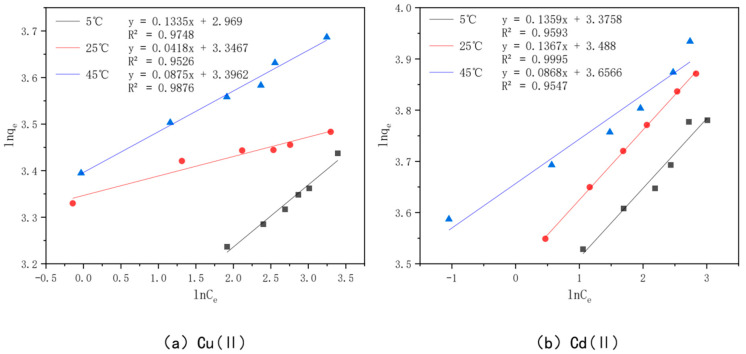
Freundlich models of KAM500-400-3 adsorbing Cu(II)/Cd(II) at different temperatures.

In order to better understand the optimal effect of modified bamboo charcoal on the adsorption of Cu(II) and Cd(II), two models, Langmuir and Freundlich, were used to fit the data obtained from the experiments. Among them, the Langmuir model satisfies the monolayer adsorption model in the ideal case, the essence of which is the assumption of the same adsorption affinity between the adsorbent and the adsorbed material. In contrast, the Freundlich model expresses the multilayer adsorption in a non-ideal situation, where the adsorbed amount of adsorbent will increase when the concentration of adsorbent increases. The Langmuir model is plotted with Ce as the horizontal coordinate and Ce/qe as the vertical coordinate, as shown in Figure 15; the Freundlich model is plotted with lnCe as the horizontal coordinate and lnQe as the vertical coordinate, as shown in Figure 16. The parameters fitted to each model are detailed in Table 7.

From the table, we see that the *R^2^* value of Cu(II) was 0.9965, 0.9996, and 0.9981 when fitted with the Langmuir model at 5 °C, 25 °C, and 45 °C, and 0.9748, 0.9526, and 0.9876 when fitted with the Freundlich model, respectively, while the *R^2^* value of Cd(II) was 0.9964, 0.9988, and 0.9955 when fitted with the Langmuir model at 5 °C, 25 °C, and 45 °C and 0.9955 when fitted with the Freundlich model, respectively. The *R^2^* values of Cd(II) were 0.9964, 0.9988, and 0.9955 for the Langmuir model at 5 °C, 25 °C, and 45 °C, respectively, and 0.9593, 0.9995, and 0.9547 for the Freundlich model, respectively. Kf in the Freundlich model represents the adsorption equilibrium concentration, i.e., the amount of adsorption per unit concentration, and the value of *n* is generally unlimited, while the value of 1/*n* is generally between 0 and 1. This represents the sensitivity of the adsorption amount to a change in solution concentration, and when 1/*n* is less than 0.5, it indicates that the adsorption process is simple. A comprehensive analysis of the values of Kf and 1/*n* shows that the value of Kf increases gradually with an increase in the adsorption temperature, which indicates that the higher the adsorption temperature, the better the adsorption capacity of KAM500-400-3 for Cu(II) and Cd(II), which is consistent with the conclusion obtained from the Langmuir model fitting, and the value of 1/*n* for Cu(II) at temperatures of 5 °C, 25 °C, and 45 °C. The values of 1/*n* were in the range of 0.04–0.13 for Cu(II) and 0.09–0.14 for Cd(II), which indicated that KAM500-400-3 could easily adsorb Cu(II) and Cd(II) [84].

The comprehensive analysis shows that the Langmuir model can more accurately describe the adsorption of KAM500-400-3 for Cu(II) and Cd(II) than the Freundlich model, indicating that the adsorption of Cu(II) and Cd(II) on KAM500-400-3 is monolayer adsorption and its main adsorption mechanism is chemisorption [85]. In addition, the *R^2^* values of the Freundlich model are also greater than 0.9, which indicates that the process of adsorption of Cu(II) and Cd(II) by the optimal effect modification is accompanied by physical adsorption in addition to chemical forces, which is consistent with the experimental results of adsorption kinetics.

### 3.8. Adsorption Thermodynamics

Substituting the experimental data into the equation, it can be seen from Table 8 that the free energy change ΔG^0^ of the adsorption of Cu(II) by KAM500-400-3 is −9.70 kJ/mol, –10.74 kJ/mol, and −12.14 kJ/mol when the temperature is 5 °C (278 K), 25 °C (298 K), and 45 °C (318 K), respectively. The values of the free energy change AG0 of the adsorption of Cd(II) were −12.70 kJ/mol, −14.27 kJ/mol, −15.65 kJ/mol, respectively. With an increase in temperature, the value of the free energy change AG_0_ of the adsorption kept decreasing and its absolute value kept increasing, indicating that the adsorption of Cu(II) and Cd(II) on KAM500-400-3 was spontaneous. The enthalpy changes of Cu(II) and Cd(II) adsorption on KAM500-400-3 are positive, 7.19 kJ/mol and 7.82 kJ/mol, respectively, which indicates that the adsorption of Cu(II) and Cd(II) on KAM500-400-3 is spontaneous, and the adsorption effect of KAM500-400-3 on heavy metals improves with increasing adsorption temperature, which is consistent with the conclusion obtained from the adsorption isotherm [86]. The 400-3 adsorption of Cu(II) and Cd(II) is heat-absorbing, increasing the temperature is beneficial for the removal of Cu(II) and Cd(II) by KAM500-400-3. Cu(II) and Cd(II) adsorption entropy changes AS on KAM500-400-3 are 60.64 J/molK and 73.93 J/molK, respectively. The adsorption process of KAM500-400-3 on Cu(II) and Cd(II) is a reaction to the entropy increase.

## 4. Conclusions

In this study, the adsorption characteristics of optimal effect modified bamboo carbon KAM500-400-3 for heavy metals Cu(II) and Cd(II) were investigated using static and dynamic adsorption experiments. The effects of the solution concentration, adsorption time, pH, and temperature on the adsorption effect of KAM500-400-3 on Cu(II) and Cd(II) were investigated in the static adsorption experiments, and the adsorption mechanism of KAM500-400-3 on Cu(II) and Cd(II) was analyzed by fitting the adsorption kinetics, adsorption isotherms, and adsorption thermodynamics. The following conclusions were obtained:

(1) The adsorption of heavy metals Cu(II) and Cd(II) by KAM500-400-3 is affected by solution concentration, adsorption time, pH, temperature, and other factors. When the initial concentration of heavy metals Cu(II) and Cd(II) increases, the adsorption amount also increases, and the relatively higher pH (pH = 6) and temperature are more favorable to the adsorption of the optimal modified effect carbon for heavy metals Cu(II) and Cd(II);
(2) The results of the adsorption kinetic fitting showed that both the proposed primary and secondary kinetics could describe the process of Cu(II) and Cd(II) adsorption by KAM500-400-3 well, but the latter was a better fit. This indicates that chemisorption and physical adsorption of Cu(II) and Cd(II) by KAM500-400-3 occur simultaneously, with chemisorption dominating. This chemisorption involves electron covalency or electron migration between the adsorbed material and the adsorbent, and physical adsorption such as precipitation and ion exchange may also exist.

The adsorption isotherm fitting results showed that the correlation coefficients *R*^2^ of both Langmuir and Freundlich models were greater than 0.9, indicating that they could both accurately describe the adsorption of KAM500-400-3 for Cu(II) and Cd(II), indicating that the process of adsorption of Cu(II) and Cd(II) by the optimal effect modification is accompanied by chemical forces. This is consistent with the experimental results of adsorption kinetics. The best fit of the Langmuir model among the two adsorption isotherm fitting models indicates that the adsorption of Cu(II) and Cd(II) on KAM500-400-3 is monolayer adsorption with chemisorption as the main adsorption mechanism.

The results of the adsorption thermodynamic fitting indicated that the adsorption of Cu(II) and Cd(II) on KAM500-400-3 was spontaneous and heat-absorbing, and the adsorption effect of KAM500-400-3 on heavy metals improved as the adsorption temperature increased, which is consistent with the conclusion obtained from the adsorption isotherm that the adsorption of Cu(II) and Cd(II) by KAM500-400-3 process is the reaction of entropy increase.

In this study, bamboo charcoal was prepared at different carbonization temperatures using Moso bamboo as a raw material, and the best carbonization temperature was investigated through adsorption experiments. Based on the shortcomings of the present study, the following work should be carried out:

(1) The biomass material selected for this experiment was Moso bamboo. In future studies, we can try to select a variety of biomass materials for carbonization and modification to investigate their adsorption capacity for Cu(II) and Cd(II) in water as well as their immobilization effect on Cu(II) and Cd(II) contaminated sediment, and to determine the optimal biomass material;
(2) The modification methods adopted in this experiment are mainly acid–base surface modification and activation modification. In future studies, we can also try redox modification by adding metal salts to biochar or adsorbent compound modification, or combine several modification methods to explore a better modification method for biochar [87];
(3) In this experiment, we only investigated the adsorption of Cu(II) and Cd(II) in the water body; we could consider the competition of heavy metals adsorption in the water body with mixed metals in a subsequent study;
(4) This experiment did not consider the regeneration and reuse of modified bamboo charcoal adsorbed with Cu(II) and Cd(II). In subsequent experiments, we may consider regenerating the contaminated modified bamboo charcoal by acid regeneration and thermal regeneration, investigate the number of times it can be regenerated, and compare the effectiveness of each method in regenerating the modified bamboo charcoal.

## Figures and Tables

**Table 1:** Adsorption mechanism of heavy metals by biochar and modified biochar.

Mechanism	Principle	Main Influencing Factors	References
Surfaceabsorption	The surface of biochar is rich in acidic groups such as carboxyl groups and phenolic hydroxyl groups, which can form specific metal complexes with heavy metal ions in water/soil and form active adsorption sites, etc.	Surface chemical bond group; Diffusion effect of heavy metal ions; Temperature.	[10,11,12,13,14]
ElectrostaticAdherence	Formation of ionic bonds (formed when atoms gain or lose electrons) between anions and cations by electrostatic interaction (chemical bonding).	Zeta potential; pH value; Degree of dispersion.	[15,16,17]
Ion Exchange	The charged cations and protons on the surface of biochar exchange with dissolved heavy metal ions in an exchange reaction.	Nature of the surface functional groups; Size of the pollutants; Live nature; pH value.	[18,19,20,21,22]
ChemicalPrecipitation	Anions react with heavy metal ions to form a water-insoluble precipitate.	pH value; Electrolyte concentration; Complexing effect; Homonymous ion effect.	[23,24,25,26]

**Table 2:** Experimental instruments.

Number	Experimental Instruments	Specifications	Use
1	Electronic balance	AUY220	Weight
2	Tube furnace	SG-GL1400	Carbonized sample
3	Constant temperature drying oven	DZ47-60	Dry sample
4	pH meter	S2211T	Measuring pH of heavy metal solution
5	Atomic absorption spectrophotometer	TAS-990SUPERAFG	Measure the concentration of heavy metal solution
6	Constant temperature culture oscillator	THZ-82	Oscillation
7	Pulverizer	LD-500A	Crushed sample
8	Vacuum pump	2XZ-4	Pump the tubular furnace into a vacuum state
9	Environmental scanning electron microscope—energy spectrometer	QUANTA200	Obtain sample surface morphology and micro area elements
10	Specific surface area analyzer	ASAP200	Aperture structure of analytical sample
11	Fourier transform infrared spectrometer	VERTEX80v	Analysis of sample surface
12	Constant-temperature water bath (HH-2)	HH-2	Heated sample

**Table 3:** Experimental reagents.

Number	Chemical Reaction	Vender	Fineness	Use
1	CuSO_4_·_5_H_2_O	Nanjing Chemical Reagents Co., Ltd.	AR	Compound Cu(II) solution
2	CdSO_4_·8/3H_2_O	MacClean	AR	Compound Cd(II) solution
3	NaOH	Xilong Science Co., Ltd.	AR	Adjust the solution pH
4	HCl	Nanjing Chemical Reagents Co., Ltd.	AR	Adjust the solution pH
5	HNO_3_	Nanjing Chemical Reagents Co., Ltd.	AR	Acid modification of bamboo charcoal
6	KOH	Sinopharm Group Chemical Reagent Co., Ltd.	AR	Bamboo charcoal was modified to alkali
7	SiO_3_	Sinopharm Group Chemical Reagent Co., Ltd.	AR	Fixed charcoal bed
8	HCl	Nanjing Chemical Reagents Co., Ltd.	AR	Nitric acid-hydrochloric acid mixture is configured
9	CH_3_COOH	Nanjing Chemical Reagents Co., Ltd.	AR	Extracted acid extracts from the heavy metals Cu(II) and Cd(II)
10	NH_2_OH·HCI	Sinopharm Group Chemical Reagent Co., Ltd.	AR	Extracting acid-recoverable heavy metals Cu(II) and Cd(II)
11	H_2_O_2_	Nanjing Chemical Reagents Co., Ltd.	AR	Extracting acid oxidation state heavy metals Cu(II) and Cd(II)
12	NH_4_COOH	Sinopharm Group Chemical Reagent Co., Ltd.	AR	A TCLP toxic leaching extract was prepared

**Table 4:** The porous structural parameters of the bamboo charcoal and the modified bamboo charcoal.

Sample Name	BET Specific Area (m^2^/g)	BJH Total Hole Capacity (cm^3^/g)	Mean Pore Size (nm)
BC500	4.2422	0.000693	20.604
KBC500-2	0.1112	—	—
KAM500-400-3	6.1656	0.003165	106.317
HBC500-30	2.8851	0.000442	20.056

**Table 5:** Element distribution table before and after the adsorption of heavy metals Cu(II), Cu(II), and Cd by bamboo charcoal and modified bamboo charcoal.

(a) BC500						
	The BC500 Was Not Adsorbed		BC500 Adsorption of Cu(II)		BC500 Adsorption of Cd(II)	
Element	Wt %	At %	Wt %	At %	Wt %	At %
C K	74.21	79.31	75.3	80.38	80.41	85.28
O K	25.79	20.69	24.42	19.57	18.23	14.57
Cu K	—	—	0.27	0.05	—	—
Cd L	—	—	—	—	1.36	0.15
Totals	100	100	100	100	100	100
**(b) KBC500-2**						
	**The KBC500-2** **Was Not Adsorbed**		**KBC500-2** **Adsorption of Cu(II)**		**KBC500-2 Adsorption of Cd(II)**	
Element	Wt %	At %	Wt %	At %	Wt %	At %
C K	69.29	75.05	73.08	79.11	70.03	76.54
O K	30.71	24.94	25.3	20.56	28.36	23.27
Cu K	—	—	1.62	0.33	—	—
Cd L	—	—	—	—	1.61	0.19
Totals	100	100	100	100	100	100
**(c) KAM500-400-3**						
	**KAM500-400-3 Was Not Adsorbed**		**KAM500-400-3** **Adsorption of Cu(II)**		**KAM500-400-3 Adsorption of Cd(II)**	
Element	Wt %	At %	Wt %	At %	Wt %	At %
C K	57.65	64.74	56.2	66.34	51.53	62.43
O K	42.35	35.26	36.02	31.92	40.05	36.48
Cu K	—	—	7.77	1.73	—	—
Cd L	—	—	—	—	8.42	1.09
Totals	100	100	100	100	100	100
**(d) HBC500-30**						
	**KAM500-30** **Was Not Adsorbed**		**KAM500-30** **Adsorption of Cu(II)**		**KAM500-30 Adsorption of Cd(II)**	
Element	Wt %	At %	Wt %	At %	Wt %	At %
C K	54.72	61.59	55.05	62.15	55.32	63.02
O K	45.28	38.41	44.54	37.76	42.98	36.77
Cu K	—	—	0.41	0.09	—	—
Cd L	—	—	—	—	1.7	0.21
Totals	100	100	100	100	100	100

**Table 6:** Fitted data of the kinetic models on optimally modified bamboo charcoal adsorbing different concentrations of Cu(II)/Cd(II) solutions.

Heavy Metal	Dynamic Model	Constant	50 mg/L	60 mg/L	70 mg/L
Cu(II)	Level 1 power	k_1_ (g/mg·h)	0.0067	0.0065	0.0092
	Learn the model	q_e_ (mg/g)	18.4	22.67	21.185
		*R* ^2^	0.8119	0.8006	0.7932
	Planned secondary power	k_2_ (g/mg·h)	0.0008	0.0003	0.0003
	Learn the model	q_e_ (mg/g)	30.49	35.97	38.02
		h_0_	0.7609	0.4198	0.4751
		*R* ^2^	0.9968	0.976	0.9695
	Intraparticle diffusion	kp_1_ (mg/g min^1/2^)	3.0492	3.9562	4.6053
	model	C_i_	−6.422	−13.015	−15.66
		*R*_1_^2^	0.9757	0.9489	0.9468
		kp_2_(mg/g min^1/2^)	0.0876	0.1229	0.0421
		C2	26.217	28.711	33.663
		*R* ^2^	0.9918	0.9762	0.975
Cd(II)	Level 1 power	k_1_ (g/mg·h)	0.0045	0.0037	0.0045
	Learn the model	q_e_ (mg/g)	3.68	3.83	2.47
		*R*_2_^2^	0.8248	0.925	0.7792
	Planned secondary power	k_2_	0.005	0.0039	0.0077
	Learn the model	q_e_ (mg/g)	42.37	47.62	48.54
		h_0_	9.0353	8.9526	18.215

**Table 7:** Fitted data from the KAM500-400-3 isotherm model adsorbing Cu(II) and Cd(II).

Heavy Metal	Temperature (°C)		Langmuir		Freundlich		
		qe	Ki	*R^2^*	Kf	*n*	*R^2^*
		(mg/g)	(L/mg)		(mg^1−1/n^·L^1/n^·g^−1^)		
Cu(II)	5	33.44	0.3646	0.9965	19.4724	7.4906	0.9748
	25	32.68	3.06	0.9996	28.4088	23.9234	0.9526
	45	40.65	1.1942	0.9981	29.8505	11.4286	0.9876
Cd(II)	5	46.95	0.6741	0.9964	29.2477	7.3584	0.9593
	25	50.25	1	0.9988	32.7204	7.3153	0.9995
	45	51.55	1.6724	0.9955	38.7294	11.5207	0.9547

**Table 8:** Thermodynamic parameters of adsorption on KAM500-400-3 absorbing Cu(II)/Cd(II).

Heavy Metal	Temperature (K)	K d	ΔG_0_ (kJ/mol)	ΔH_0_ (kJ/mol)	ΔS_0_ (kJ/mol)	*R^2^* (kJ/mol)
Cu(II)	278	33.44	−9.7	7.19	60.64	0.9582
	298	32.68	−10.74			
	318	40.65	−12.14			
Cd(II)	278	46.95	−12.7	7.82	73.93	0.9884
	298	50.25	−14.27			
	318	51.55	−15.65			

## Data Availability

Not applicable.

## Supplementary Materials

The following supporting information can be downloaded at: https://www.mdpi.com/article/10.3390/toxics10120787/s1, Experimental methods: Preparation methods of modified bamboo charcoal, Analysis methods: Analysis of surface morphology and element distribution of bamboo charcoal; Specific surface area determination; FT-IR analysis. Adsorption experiments of bamboo charcoal and modified bamboo charcoal on heavy metals Cu(II) and Cd(II). Static adsorption experiments.
