# Supplementary material for: Adsorption Characteristics of Modified Bamboo Charcoal on Cu(II) and Cd(II) in Water
Source: Toxics. 2022 Dec 14;10(12):787. doi: 10.3390/toxics10120787 (PMC9784441; doi:10.3390/toxics10120787)
Supplement: Supplementary file 1 [file toxics-10-00787-s001.zip › toxics-2033459-supplementary.pdf]

Experimental methods

1、Preparation methods of modified bamboo charcoal

(1) Preparation method of KOH-modified bamboo charcoal

Bamboo charcoal BC500 was washed with pure water and filtered by extraction, and dried in an oven at 80°C for later use[34]. 5g of washed bamboo charcoal BC500 was mixed with 100ml of KOH solution with concentrations of 0.5 mol/L, 1 mol/L, 2 mol/L and 4 mol/L, respectively, using an electronic balance, and then placed in a thermostatic shaker for 2 hours with the program setting of 25°C and 250 r/min. After shaking, the solution was filtered with pure water, washed until the pH of the solution was neutral, and then put into an oven at 80°C to dry. The obtained KOH-modified bamboo charcoal was labeled as KBC500-0.5, KBC500-1, KBC500-2 and KBC500-4 in descending order of the concentration of the KOH solution mixed with it.

(2) Preparation method of KOH activated modified bamboo charcoal

① Effect of KOH and bamboo charcoal alkali carbon ratio

5g of bamboo charcoal BC500 and 2.5g, 5g, 10g and 15g of KOH (alkali carbon ratio of 1:2, 1:1, 2:1 and 3:1, respectively) were weighed accurately with an electronic balance and mixed thoroughly in a high-temperature resistant ceramic crucible, and the program of the tube furnace was set as follows: the temperature was 700°C, the temperature was increased by 5C per minute, and the temperature was held for -hours after reaching the set temperature, and then Natural cooling, to be removed separately when cooling down to room temperature, washed with pure water to neutral and then dried in an oven at 80°C. After the preparation was completed, it was placed in a self-sealing bag and stored for later use, according to the mass of KOH added, marked in descending order as KAM500-700-0.5, KAM500-700-1, KAM500-700-2, KAM500-700- 3.

② Effect of activation temperature

The activation temperatures were set to 400°C, 500°C, 600°C and 700°C, respectively, and the tube furnace was programmed to increase the temperature by 5°C per minute, hold for hours after reaching the set temperature, and then cool down naturally. When the temperature was lowered to room temperature, they were taken out respectively, washed with pure water to neutral and then dried in an oven at 80°C. After the preparation was completed, they were put into self-sealing bags for storage for later use and labeled as KAM500-400-3, KAM500-500-3, KAM500-600-3, KAM500-700-3 in order of charring temperature from small to large.

(3) Preparation method of HNO3-modified bamboo charcoal

The bamboo charcoal BC500 was washed with pure water and extracted, and dried in an oven at 80°C for later use. 5g of washed bamboo charcoal BC500 was mixed with 100ml of HNO3 solution with mass fractions of 5%, 10%, 20% and 30%, respectively, using an electronic balance, and then put into a constant temperature shaker for 6 hours with the program setting of 25°C and 250r/min. The solution was then dried in an oven at 80°C. The nitric acid (HNO_3)-modified bamboo charcoal was labeled as HBC500-5, HBC500-10, HBC500-20 and HBC500-30 in descending order of the concentration of the HNO_3 solution mixed with it.

Analysis methods

1、Analysis of surface morphology and element distribution of bamboo charcoal

In this experiment, an environmental scanning electron microscopy-energy spectrometer model QUANTA200 was used to observe the morphological characteristics of bamboo charcoal and modified bamboo charcoal and to obtain the elemental composition of micro-regions. The principle of this instrument is to scan the sample with a fine and narrow electron beam. A small amount of powder of bamboo charcoal and modified bamboo charcoal was taken by tweezers and attached to the sample stage with conductive adhesive in a certain order. The samples were then sprayed with gold and dried. After drying, the samples were released to observe the morphological characteristics of bamboo charcoal and modified bamboo charcoal and to obtain the distribution of elemental composition in each sample.

2、Specific surface area determination

In this experiment, bamboo charcoal and modified bamboo charcoal were tested using ASAP2020 automatic specific surface area and pore analyzer BET. The BET specific surface area, pore volume and pore size of bamboo char and modified bamboo char materials were calculated by two methods, Brunauer-Emmett-Teller (BET) and Barrett-Joyner-Halenda (BJH).

$$\frac{\mathbf{P}}{\mathbf{V}(\mathbf{P_0}-\mathbf{P})}=\frac{\mathbf{1}}{\mathbf{V_m}\cdot\mathbf{C}}+\frac{\mathbf{C}-\mathbf{1}}{\mathbf{V_m}\cdot\mathbf{C}}(\mathbf{P}/\mathbf{P_0}) \qquad (1)$$

P-Gas adsorption equilibrium pressure;

P_0-saturation vapor pressure of the gas at the adsorption temperature;

V-gas adsorption volume;

V_m-monolayer molecular volume;

C-heat constant of adsorption related to the substance.

$$\mathbf{r_k = -4.14\left[\log\left(\frac{P}{P_0}\right)\right]^{-1}} \qquad (2)$$

$$\mathbf{t = -4.3\left[\frac{5}{\ln\left(\frac{P}{P_0}\right)}\right]^{-\gamma}} \qquad (3)$$

$$\mathbf{r = r_k + t}$$

$\mathbf{r_k}$-Kelvin radius;

t-thickness of the adsorption layer;

r-radius of the pores;

Γ-the surface tension of liquefied adsorbate.

3、FT-IR analysis

A VERTEX80v Fourier transform infrared spectrometer was used for the determination of functional groups on the surface of bamboo charcoal and modified bamboo charcoal. The experiment was conducted by KBr pressure method, and the whole experiment was operated under an infrared baking lamp. KBr was used as the background material, to which the sample was added (the mass ratio of KBr to sample was about 1:200), and the two were ground to 200 mesh (0.075 mm) in an agate bowl and mixed thoroughly before being placed in a tablet press and pressed at 15 MPa for 30 seconds. Then it was put into the FTIR spectrometer and scanned with a detector with a resolution of 4.0 cm-1. The scanning range was from 4000 cm-1 to 400°Cm-1d in the interval of 32 scans, and finally it was enough to keep most of the absorption peaks in the range of 10%-80% transmittance in the spectrogram.

Adsorption experiments of bamboo charcoal and modified bamboo charcoal on heavy metals Cu(II) and Cd(II)

The adsorption of heavy metals Cu(II) and Cd(II) by bamboo charcoal and modified bamboo charcoal was carried out as follows: a fixed volume and concentration of Cu(II) and Cd(II) solution was prepared, and the solution was adjusted to a fixed pH with 0.1 mol/L HCl and NaOH. After that, a certain mass of bamboo charcoal/modified bamboo charcoal was mixed with it in a 250 ml conical flask and placed in a thermostatic oscillator and shaken at 150 r/min for a certain time at a certain temperature. After the adsorption of the bamboo charcoal/modified bamboo charcoal was completed, the solution was filtered with a 0.22 pm filter head and the clear solution was collected, and a certain volume of the clear solution was fixed in a volumetric flask with dilute nitric acid (HNO3) of 0.2% concentration. The concentration of heavy metals in the solution was measured using an atomic absorption spectrophotometer, and the equations for the calculation of the adsorption and removal percentages of Cu(II) and Cd(II) are given in Equation (4) and (5).

$$\mathbf{q_t} = \frac{(\mathbf{C_0}-\mathbf{C_t})\mathbf{V}}{\mathbf{m}} \tag{4}$$

$$\mathbf{E} = \frac{\mathbf{C_0}-\mathbf{C_t}}{\mathbf{C_0}} \times \mathbf{100}\% \tag{5}$$

q_t-the adsorption amount of bamboo charcoal for Cu(II) and Cd(II) at moment t (mg/g).

C_0-initial concentration of Cu(II), Cd(II) solution (mg/L).

C_t-the concentration of Cu(II), Cd(II) in the solution at moment t (mg/L).

V-volume of Cu(II), Cd(II) solution added to the conical flask (ml).

m-mass of added bamboo charcoal(g).

E-removal rate of Cu(II), Cd(II).

1、Effect of charring temperature of bamboo charcoal

The initial concentrations and volumes of Cu(II) and Cd(II) were 50 mg/L and 120 ml, pH 6. The program settings in the thermostatic incubation shaker were: time 24 h, temperature 25°C, speed 150 r/min. The specific experimental methods were as described above, and the average adsorption of heavy metals per gram of bamboo charcoal adsorption bamboo charcoal on The removal rate of heavy metals in solution is shown in equations (4) and (5).

2、Effect of modified bamboo charcoal prepared by different methods

The initial concentrations and volumes of Cu(II) and Cd(II) were 50 mg/L and 120 ml, pH 6. The program settings in the constant temperature incubator were: time 24 h,

temperature 25°C, speed 150 r/min. The specific experimental methods were as described above. The removal rate of heavy metals in solution is shown in equations (2.1) and (2.2).

Static adsorption experiments

The control of adsorption conditions in the adsorption process has an important influence on the adsorption effect. The adsorption effect of the optimum effect modified bamboo charcoal (KAM500-400-3) on heavy metals Cu(II) and Cd(II) will change if any of the conditions are changed. This experiment mainly investigates the effects of the adsorption time, temperature during adsorption, pH and concentration of the solution on the adsorption effect of optimal effect modified bamboo charcoal (KAM500-400-3) on Cu(II) and Cd(II) in water.

1、Effect of solution concentration

The mass of KAM500-400-3 was 0.2g, the concentrations of CuS04 and CdSO4 were 50mg/L, 60mg/L and 70mg/L respectively, the volume of CuS04 was 150ml, the volume of CdSO4 was 180ml, the pH was adjusted to 6, and the program settings in the constant

temperature incubator were: time 24 hours, temperature 25°C, rotation speed 150r/min. The average amount of heavy metals adsorbed per gram of KAM500-400-3 and the removal rate of heavy metals from the solution are shown in equations (4) and (5) .

2、Experiment on the effect of solution pH

The mass of KAM500-400-3 was 0.1g, the concentration of CuS04 and CdSO4 was 50mg/L, the volume of CuS04 was 75ml, the volume of CdSO4 was 90ml, the pH was adjusted to 2, 3, 4, 5 and 6 respectively, and the program settings in the constant temperature culture shaker were: time 24 hours, temperature 25C, speed 150r/min. The average amount of heavy metals adsorbed per gram of KAM500-400-3 and the removal rate of heavy metals from the solution were shown in equations (4) and (5) . The above adsorption experiments at each pH were repeated three times and the final results were taken as the average of the three values.

3、Effect of adsorption time and adsorption kinetics

The mass of KAM500-400-3 was 0.2g, the concentrations of CuS04 and CdSO4 were 50mg/L, 60mg/L and 70mg/L respectively, the volume of CuS04 was 150ml, the volume of CdSO4 was 180ml, the pH was adjusted to 6, and the program settings in the constant temperature culture shaker were: time 24 hours, temperature 25C, speed 150r/min. /Samples were taken at adsorption times of 10 min, 20 min, 30 min, 60 min, 90 min, 120 min, 240 min, 360 min, 480 min, 1440 min (24 h), and the average amount of heavy metals adsorbed per gram of KAM500-400-3 and the removal rate of heavy metals from the solution were shown in Equation (4) and (5).

As Eq. (6) is a quasi primary kinetic model equation :

$$\ln(q_e - q_t) = \ln q_e - k_1 t \tag{6}$$

Equation (7) is a quasi-secondary kinetic model equation:

$$\frac{dq}{dt} = k_2(q_e - q_t)^2 \tag{7}$$

By setting t and q to 0 as the boundary, we can introduce:

$$\frac{1}{q_t} = \frac{1}{q_e} + k_2 t \tag{8}$$

$$\frac{t}{q_t} = \frac{1}{k_2 q_e^2} + \frac{t}{q_e} \tag{9}$$

$$h_0 = k_2 q_e^2 \tag{10}$$

Equation (11) is the intra-particle spreading model equation:

$$q_t = k_p t^{\frac{1}{2}} + C \tag{11}$$

q_e-equilibrium adsorption of heavy metals by KAM500-400-3 (mg/g)

k_2 - rate constant of quasi-secondary kinetic equation (g/mg・h).

h_0 - the initial rate of heavy metal adsorption by KAM500-400-3.

k_p-is the intraparticle diffusion rate constant (mg/gmin1/2).

C- the thickness of the boundary layer.

4、Adsorption isotherm

The mass of KAM500-400-3 was 0.1g, the concentrations of CuS04 and CdSO4 were both 50mg/L, the pH was adjusted to 6, the volumes of CuS04 were 60ml, 70ml, 80ml, 90ml, 100ml, 160ml, and the volumes of CdSO4 solution were 70ml, 80ml, 90ml, 100ml, 120ml, 140ml. The program settings in the thermostatic incubator were: time 24 h, temperature 5°C, 25°C, 45°C, speed 150 r/min. The specific experiments were performed as described in 2.2.5. The average amount of heavy metals adsorbed per gram of KAM500-400-3 and the removal rate of heavy metals from the solution were shown in equations (4), (5).

The obtained data are fitted with Langmuir and Freundlich models respectively, and the equations of these three models are shown in equations (12)-(15):

$$\mathbf{q_e = \frac{k_i q_m C_e}{1 + k_i C_e}} \tag{12}$$

The linear expressions are as follows:

$$\mathbf{\frac{C_e}{q_e} = \frac{C_e}{q_m} + \frac{1}{k_l q_m}} \tag{13}$$

$$\mathbf{q_e = k_f C_e^{\frac{1}{n}}} \tag{14}$$

$$\mathbf{\ln q_e = \ln k_f + \frac{1}{n} \ln C_e} \tag{15}$$

q_e - adsorption amount of adsorbed heavy metals.

q_m-Theoretical saturation adsorption amount;

k_i-Langmuir equation parameter (L/mg).

C_e-Equilibrium concentration of heavy metals Cu(II) and Cd(II) adsorbed by KAM500-400-3 (mg/L).

k_f-Freundlich equation parameter ($mg^{1-1/n} \cdot L^{1/n} \cdot g^{-1}$).

n-constant related to the adsorption intensity.

(5) Adsorption thermodynamics

The mass of KAM500-400-3 was 0.1 g, the concentration of CuS04 and CdSO4 was 50 mg/L, the pH was adjusted to 6, the volume of CuS04 was 160 ml, and the volume of CdSO4 was 140 ml. The program settings in the thermostatic incubator were: time 24 h, temperature 5°C, 25°C, 45°C, and speed 150 r/min, respectively. The average amount of heavy metals adsorbed per gram of KAM500-400-3 and the removal rate of heavy metals from the solution are shown in equations (4) and (5).The effect of temperature on the adsorption of Cu(II) and Cd(II) by KAM500-400-3 can be described by the adsorption

thermodynamic equation and the relationship between the parameters ΔG_0, ΔS_0, ΔH_0 is given in the following equation, and the method of calculating Kd is given in Eq.

$$\mathbf{\Delta G^0 = -RTlnK_d = \Delta H^0 - T\Delta S^0} \quad (16)$$

$$\mathbf{lnK_d = -\frac{\Delta H^0}{RT} + \frac{\Delta S^0}{R}} \quad (17)$$

Kd - thermodynamic equilibrium constant;

ΔG_0 - free energy of adsorption (J/mol);

R-ideal gas constant 8.314 J/(mol^{-K});

T - thermodynamic temperature (K);

ΔH_0- enthalpy change of adsorption (J/mol);

ΔS_0-entropy change of adsorption (J/mol·K);

q_c - amount of heavy metal adsorbed at equilibrium (mg/g);

C_e-heavy metal concentration at equilibrium (mg/L).
